# History of a Sarcomatous Tumor, Its Extirpation, &c.

**Published:** 1831

**Authors:** Washington W. Hitt

**Affiliations:** Vincennes, Indiana


Art. II.—*History of a Sarcomatous Tumor, its extirpation, &c.*—Published by request of the Medical Board of the First Medical District of Indiana.—By Washington W. Hitt, M. D., of Vincennes, Indiana.

The subject of this history is a Mrs. Holmes, of this place, aged 24 years. The previous history she gave me of her affliction is as follows:—About twelve years ago, when her system commenced its expansion, she discovered her left breast gradually to increase in size, and to continue to grow after the system had received its ultimate development; giving her no pain, and consequently no uneasiness or alarm, until after she was united in marriage to her present husband. During pregnancy she thought it increased more rapidly; and after she was delivered of her first born, its increase became more evident and alarming. A few days after the birth of the child, the gland had every appearançe, as she thought, of having secreted milk; and so much distended, that medical advice was thought necessary, and was obtained.

The child was denied access to the enlarged breast, and stimulating applications prescribed with the view to suspend the secreting process. About this time she thinks a redness appeared, circumscribed to about the size of half a crown, one inch to the right of the nipple; which redness or inflammation soon gave place to suppuration and the discharge of a fluid, the color and consistency of which she does not now recollect. After discharging a few days, it entirely healed, and no perceptible change was discovered until the second accouchment. Between her first and second pregnancy her medical attendant, she states, was as assiduous as unsuccessful in his endeavors to discuss the tumor, by the exhibition of medicine internally and externally, until ptyalism was produced and continued for some time.

On Christmas last, she was delivered of her second and last child, when the tumor again resumed its progressive march, and by its accelerated action and unyielding disposition, produced in the mind of the patient the most awful apprehensions.

The following April, I first saw her, having been called to relieve her of an attack of fever; at which time the tumor was shown to me as a curiosity. And what will be thought surprising, in a subsequent part of this history, the child was then applied to the enormous breast, which, from its magnitude, looked very unlike the mamma. I then had ocular demonstration of the fact, that secretion of milk was performed; and the child's healthful aspect itself proved it to be amply supplied with the pabulum of life. The tumor at this time was not particularly examined, as it was not requested.

I did not see the patient again until the first of July following. She was then much alarmed in consequence of a circumscribed redness, the size of a crown, appearing about four inches below the nipple; and which having subsided, left, to the feel of the finger, evident fluctuation. The external appearance of the tumor was smooth, uniform, and elastic; possessing little sensibility, and slight soreness on pressure. Upon pressing the sides of the tumor with the fingers, the internal structure imparted an uneven and irregular feeling. Its largest circumference measured 32 inches, and its base 22. Its supposed weight, from 15 to 20 pounds. It was supported by a bandage from the neck, assisted by her left arm and hand, which were constantly applied beneath it. None of the neighboring glands were implicated.

Considering the circumstances of the case, the age and comparatively good constitution of the patient, I gave opinion that extirpation was not only advisable, but would be the only possible means of her salvation from an untimely grave. She did not fully determine to submit to an operation until the above opinion was corroborated by the opinions of several gentlemen now present, who did me the kindness to examine the tumor. Her mind being fully made up, the 20th of July was appointed for the operation. From an unyielding desire to undergo the painful ordeal among her nearest kindred, from which she could not be dissuaded, she was conveyed in a carriage 22 miles north of this place, a few days previous.

Every preparation being made, my friend Dr. Somers and myself, set off on the 19th, and on our way met an acquaintance just from the patient, who stated that nature had anticipated us, and had performed the operation by the tumor's bursting and discharging. We went on however, and found the tumor collapsed, and still discharging gradually a fluid, the color and consistency of which resembled thick, rich cream.

Judging from the vessels containing the fluid, and also the condition of her clothes, there must have been at least one gallon discharged. The fluid had a pulpy feel, and peculiar smell; not offensive however. The probe could be plied in several directions, and in some it was not of sufficient length to determine its boundaries. The minds of the patient and her friends were much relieved, supposing the late occurrence would supercede the necessity of an operation. And in vain did we endeavor to convince them otherwise. We deemed it useless to insist on an operation under existing circumstances, but contented ourselves with controverting the notion, that its discharge was a sufficient reason to delay the operation; and told them it was probable she would be placed under less favorable circumstances for an operation when it would be absolutely necessary for one to be performed. After expressing our views, we passed on to Terre Haute. While there, our patient was taken with a remittent fever, and during the second exascerbation of fever, the blood vessels of the tumor gave way, and an alarming hæemorrhage ensued. A messenger was immediately dispatched, and we were hurried back to the patient. July 25th, found hor much exhausted from fever and loss of blood; and still bleeding.

In consultation with Drs. Somes, Davis, Ohowver, and Elliot, it was agreed that an operation was the only possible expedient to save her from a speedy dissolution; and that even that was extremely doubtful under existing circumstances—such as her great debility, considerable fever, the quantity of blood she had already lost, her mind agitated with fearful apprehensions, and the tumor collapsed into a loose mass, all tending to an unfavorable prognosis. By the assistance of the above-named gentlemen, the operation was commenced in the usual way of extirpating cancerous breasts—by making two semi-circular incisions; dissecting back a sufficiency of the integuments to cover the exposed surface after the removal of the tumor. The tumor was then cautiously dissected from above downward; great care being taken to tie the arteries as they were exposed. Thus the whole tumor was carefully and entirely removed, leaving the pectoral muscle bare. The patient was then laid on the bed to recover from syncope, the flaps lying open and the whole covered with lint.

I would here state that our patient was remarkably courageous, and bore the operation with a fortitude seldom witnessed in the most extensive operating theatres. She was supported with wine and water as occasion required. After she had somewhat revived, and finding no hæmorrhage on raising the lint, the flaps were properly adjusted and retained by adhesive straps. The compress and bandage completed the dressings. She was then cheered, encouraged, and congratulated, which elicited from her a smile prospective of returning health; and all faces beamed with gratitude and thankfulness for such a deliverance.

In a few days her fever subsided; the healing process commenced, and kindly progressed. Three-fourths of the flaps healed by the first intention; and in three weeks from the day of operation, she returned to her domestic circle; when three weeks more completed the entire healing of the wound.

Viewing her situation previous to the operation, when she was prostrate beneath the arm of disease, and to all appearance on the verge of dissolution, and seeing her now, reinstated to health, family, and friends, we cannot but consider this case a striking illustration of the powerful influence of our art in ameliorating the condition of man, aad a conclusive confutation of the vulgar prejudices entertained against it by some.

*Dissection of the Tumor.*—The tumor was opened by cutting through a thick capsule, made apparently of condensed cellular substance. On its surface next the skin, the capsule firmly adhered. The substance of which the principal part of the tumor was composed, was made up of irregular-shaped masses, in color and texture somewhat resembling the masses which compose the pancreas; and appeared to be connected with each other by a fibrous substance of a looser texture. A very small portion extending from the nipple to the clavicular margin of the tumor, bore the appearance of healthy gland. This was half an inch thick at the nipple, and gradually increased in thickness as it ascended to the margin; and composed, perhaps, one-twelfth part of the whole, separate from the fluid.

It is remarkable in this case, considering how small a portion of the whole mamma was in a healthy state, and shaped as it was, that milk was pressed from the nipple only two weeks previous to the operation. The cavity of the tumor occupied its centre, extending principally below, and three or four inches above the centre.

